# Tumoral calcinosis of unusual location in a chronic hemodialysis patient

**DOI:** 10.1259/bjrcr.20220083

**Published:** 2023-02-15

**Authors:** Youssef Sakhy, Reda Taoussi, Vianney Ndayishimiye, Mouna Sabiri, Mohammed Labied, Ghizlane Lembarki, Samia El Manjra, Samira Lezar, Fatiha Essodegui

**Affiliations:** 1 Central Radiology Department, Ibn Rochd University Hospital, Casablanca, Morocco; 2 Emergency Radiology Department, Ibn Rochd University Hospital, Casablanca, Morocco

## Abstract

Tumoral calcinosis is a rare cause of intratissular calcifications in hemodialysis patients with chronic renal failure. Its frequency is estimated between 0.5 and 7% of patients. We illustrate through a case of unusual localization diagnosed in Ibn Rochd University Hospital, Casablanca, Morocco, the radiographic and scannographic aspect of this little known entity. A 40-year-old man, followed for hypertensive cardiopathy, in chronic renal failure for 12 years under hemodialysis, consulted for bilateral inguinal swellings evolving in a progressive and painless way. Biological investigations revealed hyperparathyroidism with increased phosphocalcic product. He was referred to us for radiological evaluation which revealed lesions in favor of bilateral puboinguinal tumor calcinosis. Tumoral calcinosis is a rare cause of intratissular calcifications in chronic renal failure patients undergoing hemodialysis. Pubic localization with infiltration and osteolysis of the symphysis pubis is very rare. Its main risk factors are the existence of hyperparathyroidism, an increase in phosphocalcic product and probably local traumatic factors. Tumoral calcinosis has a typical appearance on radiographs: amorphous, cystic and multilobulated calcifications of periarticular distribution. The CT scan allows a better delineation of the calcified mass. Its treatment remains controversial. The knowledge of osteoarticular manifestations of chronic hemodialysis patients, especially tumoral calcinosis by radiologists, allows to easily make the diagnosis and thus avoid invasive complementary explorations for the patient and to quickly institute an effective treatment.

## Introduction

Tumoral calcinosis is a condition characterized by the deposition of calcium phosphate crystals in the periarticular soft tissues, resulting in large calcified masses up to 20 cm in diameter.

Its etiologies are diverse:^1^ autosomal dominant genetics with variable penetrance (formerly considered “idiopathic”), primary or secondary hyperparathyroidism on chronic renal failure, vitamin D intoxication, scleroderma, dermatomyositis, sarcoidosis...).

Whatever the etiology, the role of hyperphosphatemia with increased phosphocalcic product seems to be a determining factor in the pathophysiology. The role of repeated joint microtrauma has also been suggested.^1^

In dialysis patients, the frequency of tumoral calcinosis is estimated to be between 0.5 and 7% depending on the series.^1^

We report a case of tumoral calcinosis of unusual localization in a hemodialysis patient.

## Clinical observation and iconography

A 40-year-old male, followed for hypertensive heart disease, in chronic end-stage renal failure for 12 years on hemodialysis, consulted for bilateral, painless inguinal swellings, progressively increasing in volume for 1 year.

The skin was normal at the site of the lesion, with no inflammatory changes. A clinical and biological examinations were performed (Table 1).

**Table 1.** Patient’s clinical and biological parameters

Hip mobility	Serum phosphorus	Serum calcium	PTH	Size of lesion before treatment	Size of lesion after treatment
Limited painful abduction : 25° Limited flexion : 90° Free movement angles otherwise	24 mg l^−1^ 0.7 mmol l^−1^	120 mg l^−1^ 2.99 mmol l^−1^	867 pg ml^−1^ (*N* < 53)	Approx. 14 × 10 cm	Approx. 10 × 5 cm (5 months after surgery)

PTH, parathyroid hormone.

**Figure 1.**
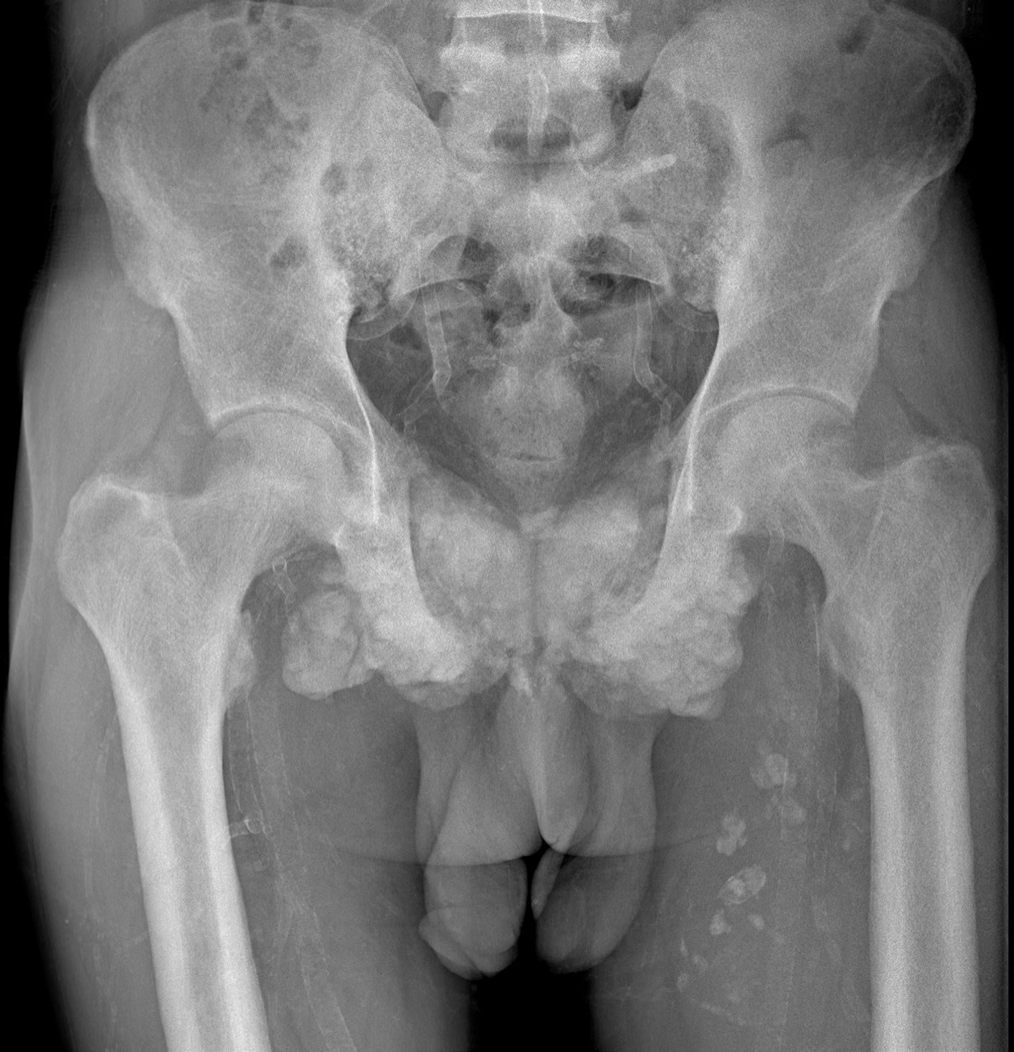
Frontal radiograph of the pelvis showing a predominantly calcified medial structure projecting onto the pubic symphysis with calcifications of the soft parts of the left thigh and diffuse mediacalcosis (Figure 1).

The standard radiograph of the pelvis showed a predominantly calcified medial structure projecting onto the pubic symphysis with calcifications of the soft parts of the left thigh and diffuse mediacalcosis (Figure 1).

**Figure 2.**
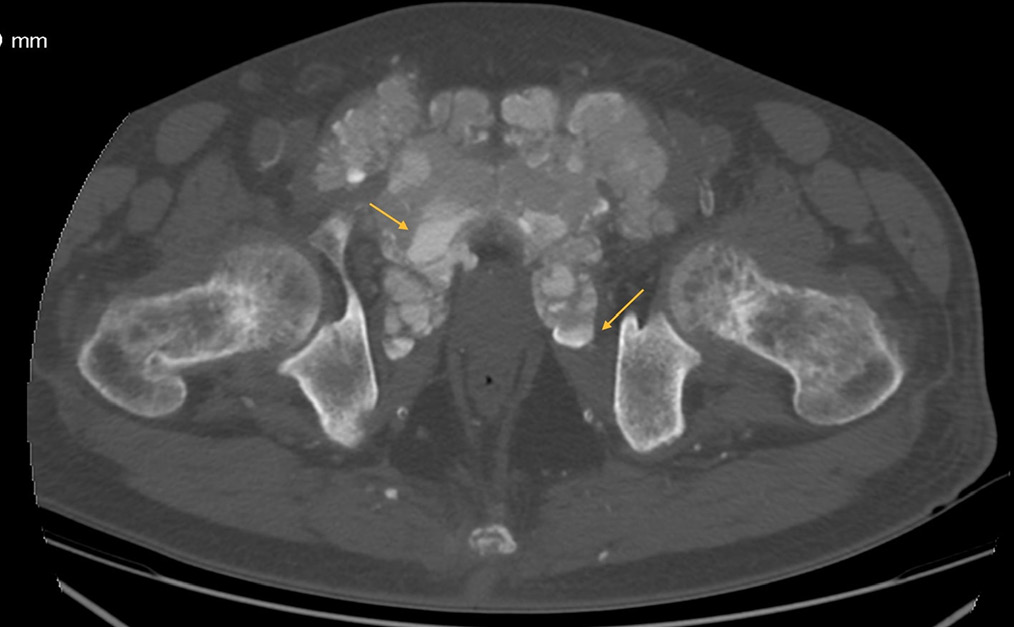
CT scan of the pelvis in axial sections showing bilateral, dense, multilobulated periarticular masses with sedimentation sign (arrows).

**Figure 3.**
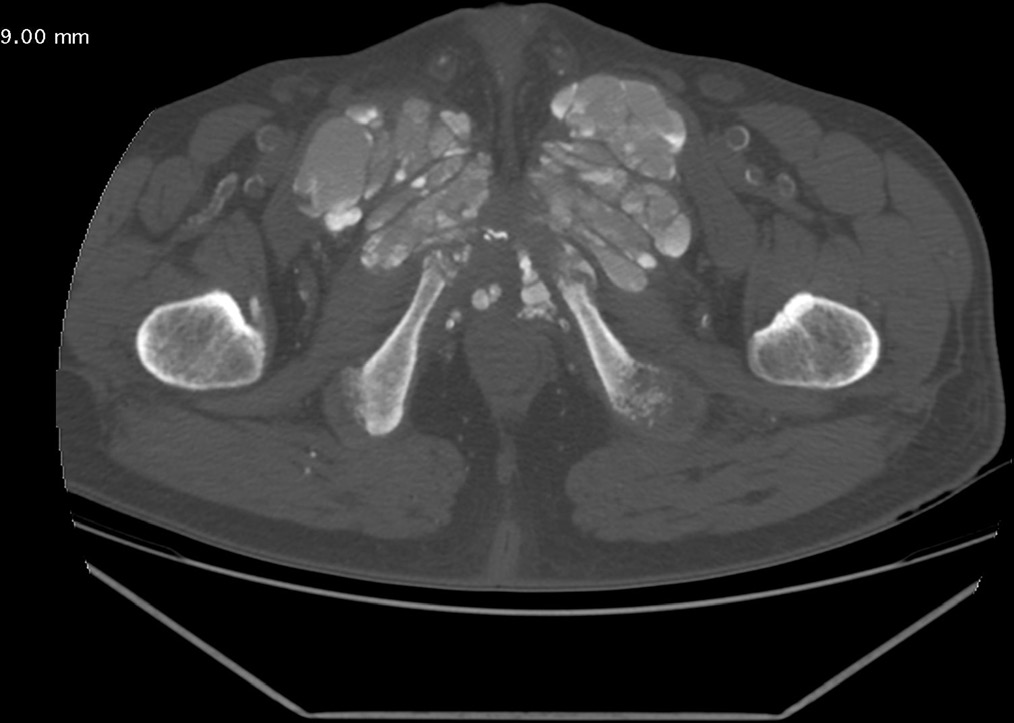
CT scan of the pelvis in axial sections showing bilateral, dense, multilobulated periarticular masses with bone erosion opposite.

Pelvic CT scan showed a bilateral symmetrical mass, centered on the pubic symphysis, with amorphous, dense cystic and lobulated calcifications, measuring approximately 14.4 × 9.9 cm, extending over 9.4 cm with pubic osteolysis and massive infiltration of the adjacent adductor muscles (Figures 2 and 3).

**Figure 4.**
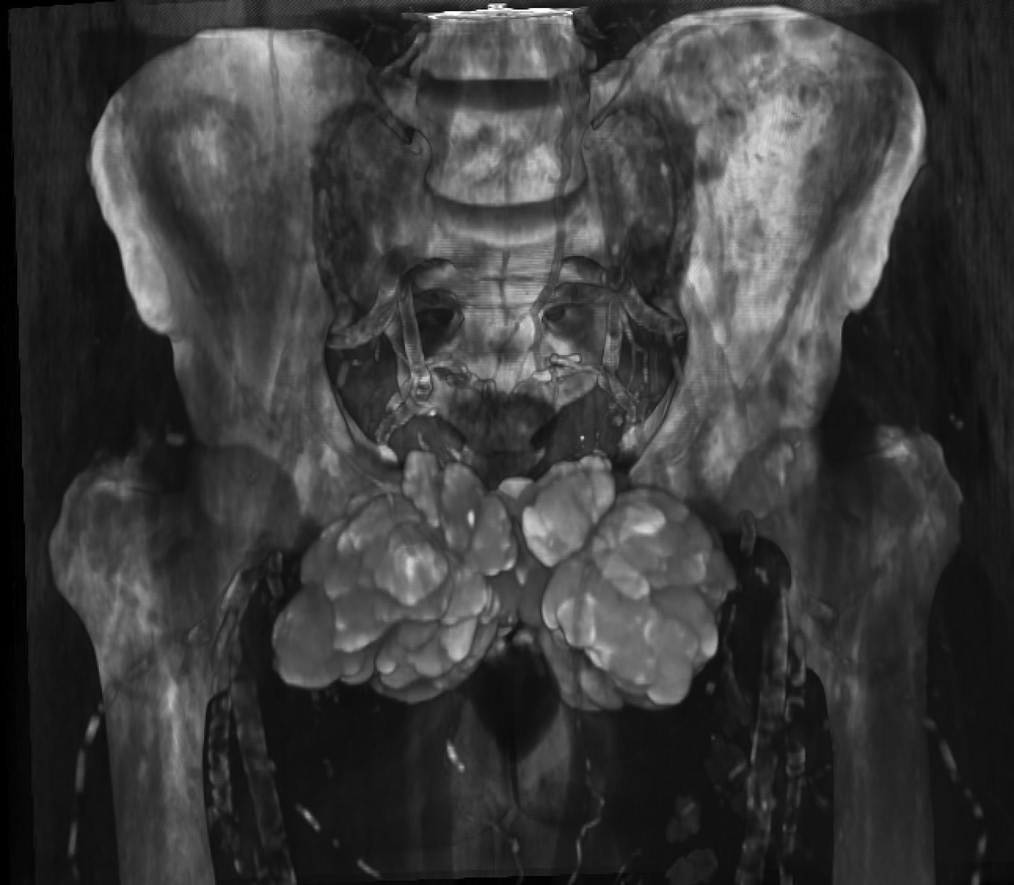
CT scan of the pelvis in 3D reconstruction showing bilateral and symmetrical puboinguinal masses. 3D, three-dimensional.

Some of the cystic masses had liquid–liquid levels with calcium sedimentation (Figure 2), the 3D reconstructions better show the extent of the disease (Figure 4).

The diagnosis of tumoral calcinosis was retained. The therapeutic management included first measures to regulate the phosphocalcic balance and then the indication of a parathyroidectomy was justified by the secondary hyperparathyroidism and the presence of parathyroid hyperplasia on scintigraphy.

The patient benefitted from a total parathyroidectomy with partial regression of his symptoms.

**Figure 5.**
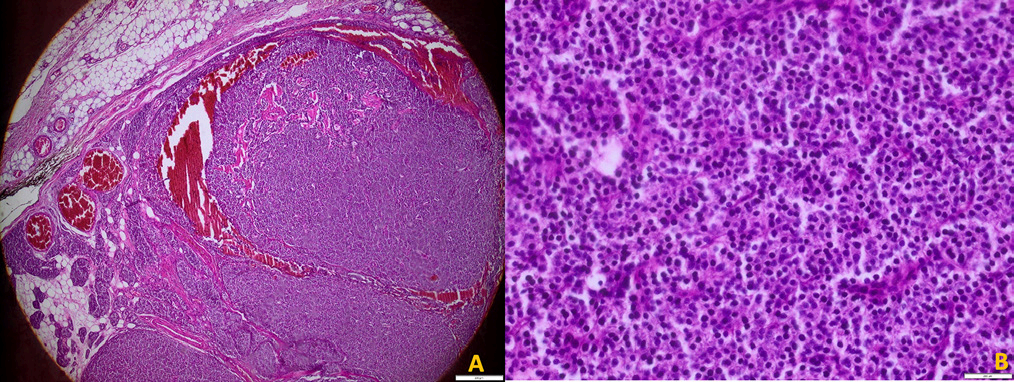
Benign tumor proliferation, well limited, nodular and lobular in architecture, covered by a thin capsule (Photo A, HE, **x4**). This proliferation is made of chief cells, without atypia and without mitoses (Photo B, HE, x40).

Pathology findings are depicted on Figure 5.

Informed consent for the case to be published was obtained directly from the patient.

## Discussion

Tumoral calcinosis should not be confused with Teuschlander’s tumoral calcinosis, also known as Inclan’s calcinosis,^1^ which, although long classified as an idiopathic calcinosis, is now considered a genetic disease.

In the literature, the average time of appearance of the mass after the start of dialysis seems to be very variable, from a few months to several years.^2^ Clinically, the lesions present as pseudotumor masses of usually slow evolution, up to 20 or 30 cm in diameter. They are most often located in the vicinity of large joints (hips, knees, shoulders, elbows) and sometimes on the extremities.^2^ Pubic involvement is rare. The skin is usually normal in front of the lesions, which are usually asymptomatic.^1^ Much more rarely, they may fistulate to the skin, limit joint movement, or cause nerve or vascular compression. Lysis of the surrounding bone structures by calcinosis is exceptionally reported.^3^ The observation of tumoral calcinosis presented here is therefore original because of its location, but also because of its local aggressiveness (pubic osteolysis).

Although the pathophysiology is not fully elucidated, the increase of the phosphocalcic product beyond the precipitation threshold (>70), more than severe hyperparathyroidism, seems to play a determining role.^4^ Indeed, several cases of tumoral calcinosis have already been reported without associated hyperparathyroidism or after parathyroidectomy.^4^ In patients with chronic renal failure, there is a defect in phosphorus filtration responsible for an increase in intracellular phosphates and hyperphosphatemia. Hypocalcemia is associated with a relative deficit in vitamin D, mainly due to a defect in renal 1-hydroxylation. These disorders (hypocalcemia and hyperphosphatemia) stimulate the production of parathyroid hormone, leading to secondary hyperparathyroidism.

On radiographs, the lesion is multilocular, para-articular with clustered juxtaposition of small dense rounded or oval images, well limited by a ring, with a more or less opaque homogeneous content depending on the density of calcium crystals and sometimes a liquid level within an elementary formation related to the sedimentation of calcium crystals with serous supernatant (sedimentation sign). Erosions of the bone cortex adjacent to the calcinosis are rarely described. The CT scan shows the local extension of the tumor.^5,6^

The ultrasound appearance has been poorly described in the literature; the lesions present as multiloculated masses with a calcified peripheral shell and fluid content.

**Figure 6.**
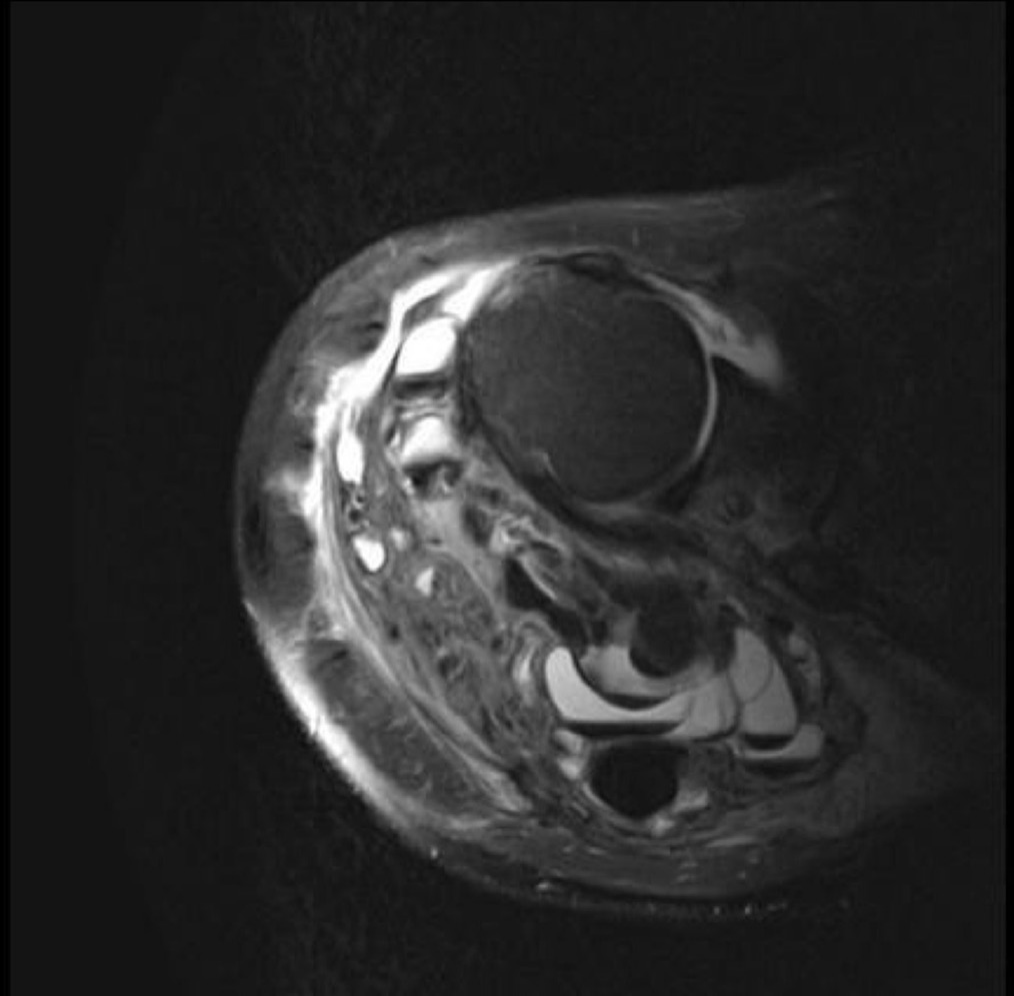
Axial *T*_2_ weighted fat suppressed images of the shoulder in another patient revealing a periarticular, multilobulated mass with multiple fluid–fluid levels. The mass is well-demarcated, lobulated and contains low signal layering “milk of calcium”. Case courtesy of Ayaz Hidayatov, <a href=“https://radiopaedia.org/“>Radiopaedia.org</a>. From the case <a href=“https://radiopaedia.org/cases/52845”>rID: 52845</a>.

On MRI, in *T*_1_ weighted sequences, tumoral calcinosis appears mostly hypointense due to the calcific nature of the tumor and the peritumoral edema, and in T2 predominates a nodular heterogeneous hyperintensity related to the inflammatory reaction associated with areas of low intensity reflecting calcium deposits (Figure 6).

Bone scintigraphy allows the early detection of these deposits and the assessment of their progressive nature.^6^

Destruction of the adjacent bone may be the result of erosion due to repeated microtrauma to the bone caused by the large size of the mass and its periarticular location.

In the presence of radiographic evidence of bone destruction, a neoplasia such as chondrosarcoma should be included in the differential diagnosis. A sedimentation sign, most often appreciated on CT scan, if detected, is helpful in establishing the diagnosis of tumoral calcinosis.^7^

The treatment of tumoral calcinosis is difficult. Surgical removal of the calcified mass is controversial. It seems to be indicated mainly in compressive forms. When it is performed, it must be as complete as possible in order to avoid recurrence.

Medical treatment aimed at balancing the phosphocalcic product is essential (phosphorus binders, hypoprotein diet, dialysis baths low in calcium).

When secondary hyperparathyroidism is associated, parathyroidectomy can give spectacular results.^1^ However, tumoral calcinosis has been observed in subjects who have previously undergone parathyroidectomy,^1^ suggesting that such a procedure should only be considered for severe hyperparathyroidism with normo or even hypercalcemia and very high parathyroid hormone.^8^ Dramatic regression of PTC after renal transplantation has also been reported.^1^

The existence of this rare condition seems to us to be important to recall, in order to evoke it rapidly in a chronic hemodialysis patient in front of any deep cutaneous lesion of tumoral appearance, alternatives to surgery being possible and most often desirable.

## Conclusion

Osteoarticular complications are frequent and disabling during end-stage renal disease.

Patients with tumoral calcinosis usually present with periarticular masses associated with pain and limitation of joint motion usually affecting the hip, elbow and shoulder.

Diagnosis is difficult with imaging alone and must be based on a combination of typical radiological features and biochemical profile.

Knowledge of the osteoarticular manifestations of chronic hemodialysis patients, especially tumoral calcinosis, by radiologists, allows for easy diagnosis and thus avoid invasive additional investigations for the patient, and for rapid initiation of effective treatment.

## Learning points

- Tumoral calcinosis is a rare entity and should be considered in the differential for osteoarticular manifestations of chronic hemodialysis patients.
- Pubic symphysis is an unusual location for tumoral calcinosis.
- Knowledge of this entity allows for an early diagnosis and better management ths reducing morbidity.
